# Bispecific radioimmunoconjugates exploit receptor heterogeneity for positron emission tomography of tumors expressing HER2 and/or EGFR

**DOI:** 10.1016/j.isci.2024.109750

**Published:** 2024-04-15

**Authors:** Luke Yongkyu Kwon, Zhongli Cai, Azza Al-Mahrouki, Raymond M. Reilly

**Affiliations:** 1 Department of Pharmaceutical Sciences, University of Toronto, Toronto, ON M5S 3M2, Canada; 2 Department of Medical Imaging, University of Toronto, Toronto, ON M5T 1W7, Canada; 3 Princess Margaret Cancer Centre, University Health Network, Toronto, ON M5G 2M9, Canada

**Keywords:** cancer, Nuclear medicine, Therapeutics

## Abstract

HER2 heterogeneity is a challenge for molecular imaging or treating HER2-positive breast cancer (BC). EGFR is coexpressed in some tumors exhibiting HER2 heterogeneity. Bispecific radioimmunoconjugates (bsRICs) that bind HER2 and EGFR were constructed by linking trastuzumab Fab through polyethyleneglycol (PEG_24_) to EGF. We established s.c. tumors in NOD-SCID mice that homogeneously or heterogeneously expressed HER2 and/or EGFR by the inoculation of HER2-positive/EGFR-negative SK-OV-3 cells, EGFR-positive/HER2-negative MDA-MB-468 cells or mixtures of these cells. [^64^Cu]Cu-NOTA-trastuzumab Fab-PEG_24_-EGF were compared to [^64^Cu]Cu-NOTA-trastuzumab Fab or [^64^Cu]Cu-NOTA-EGF for the PET imaging of HER2 and/or EGFR-positive tumors. [^64^Cu]Cu-NOTA-trastuzumab Fab-PEG_24_-EGF bsRICs imaged tumors expressing HER2 or EGFR or heterogeneously expressing these receptors, while monospecific agents only imaged HER2-or EGFR-positive tumors. Our results indicate that bsRICs labeled with ^64^Cu are able to exploit receptor heterogeneity for tumor imaging. PET may select patients for radioimmunotherapy with bsRICs complexed to the β-particle emitter, ^177^Lu or Auger electron-emitter, ^111^In in a theranostic approach.

## Highlights

• Tumors homogeneously or heterogeneously expressing HER2 and/or EGFR in NOD-SCID mice
• Bispecific radioimmunoconjugates imaged tumors heterogeneous for HER2 and EGFR
• Monospecific radioimmunoconjugates only imaged tumors homogeneous for HER2 or EGFR

## Graphical abstract

**Figure:**
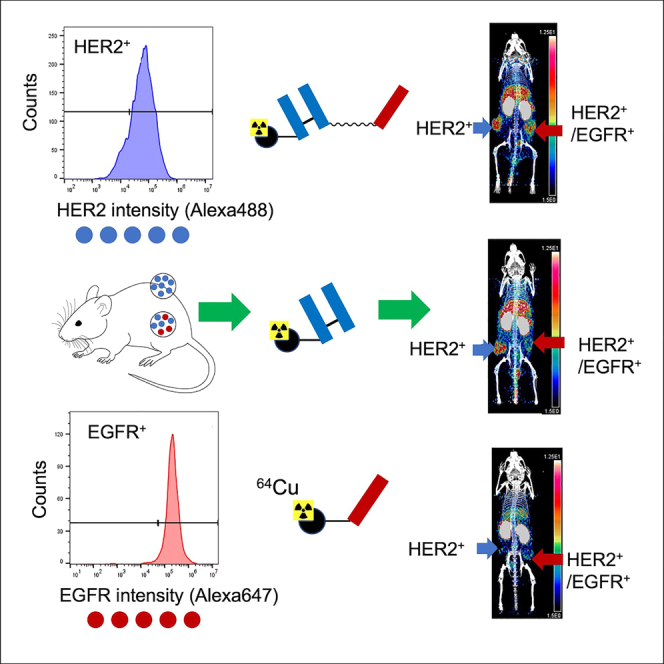

## Introduction

The human epidermal growth factor receptor (HER2) is overexpressed on 15–20% of breast cancers (BC)^1^ and is the therapeutic target for trastuzumab (Herceptin, Roche), pertuzumab (Perjeta, Roche) and the antibody-drug conjugate (ADC) trastuzumab-emtansine (T-DM1; Kadcyla, Roche).^2^ HER2 status is assessed on a tumor biopsy by immunohistochemical (IHC) staining for HER2 protein on the cell membrane or by *in situ* hybridization (ISH) to identify *HER2* gene amplification.^3^ Only patients with HER2-positive BC are selected for HER2-targeted therapies, although it has been reported that patients with BC exhibiting low (IHC 1+) or moderate HER2 expression (IHC 2+) without *HER2* gene amplification may benefit from treatment with trastuzumab deruxtecan, a potent ADC.^4^ Nonetheless, despite the careful application of criteria to define HER2-positivity, heterogeneous intratumoral HER2 staining and/or *HER2* gene amplification with HER2-positive and HER2-negative regions within the same tumor have been reported in 6–36% of tumors scored as HER2-positive.^5^ HER2 heterogeneity is correlated with a poor response to HER2-targeted therapies including trastuzumab,^6^ trastuzumab and pertuzumab combined with chemotherapy^7^ and trastuzumab emtansine (T-DM1).^8^ HER2 receptor heterogeneity has further been correlated with shorter disease-free survival in patients with HER2-positive BC.^9^

One strategy to overcome and exploit receptor heterogeneity in HER2-positive BC may be to target both HER2 and epidermal growth factor receptors (EGFR). HER2 forms heterodimers with EGFR that initiate a strong tumor growth signal.^10^ This is the rationale for combining pertuzumab, an inhibitor of HER2 dimerization, with trastuzumab for the treatment of HER2-positive BC.^11^ In one study of 119 surgical tumor specimens, 14% of tumors that were classified as HER2-positive were EGFR-positive.^12^ Coexpression of HER2 and EGFR increased the risk for metastasis and was associated with shorter disease-free and overall survival (OS). In another study of 2,567 tumors, EGFR positivity was found in 18% of cases, but EGFR-positive BC was more likely to be HER2-positive, with 26% of EGFR-positive tumors HER2-positive.^13^ Furthermore, EGFR/HER2 heterodimerization has been proposed as a mechanism of resistance to HER2-targeted therapies, since trastuzumab does not block the dimerization of HER2 with the EGFR.^14^ To exploit receptor heterogeneity in HER2-positive BC, we previously reported bispecific radioimmunoconjugates (bsRICs) that recognize HER2 and EGFR.^15^ These bsRICs were composed of trastuzumab Fab linked through a 24-mer polyethyleneglycol (PEG_24_) spacer to EGF and modified with diethylenetriaminepentaacetic acid (DTPA) to complex ^111^In [t_1/2_ = 2.8 days; Eγ = 171 keV (90.7%) and 245 keV (94.1%)]. At 48 h post-injection (p.i.) of [^111^In]In-DTPA-trastuzumab Fab-PEG_24_-EGF bsRICs, subcutaneous (s.c.) SK-OV-3 human ovarian cancer xenografts that were HER2-positive but EGFR-negative, MDA-MB-231 human BC xenografts that were HER2-negative but EGFR-positive, and MDA-MB-231/H2N tumor xenografts that coexpressed HER2 and EGFR were imaged in mice by single photon emission computed tomography (SPECT). Furthermore, in a subsequent study, radioimmunotherapy (RIT) with [^111^In]In-DTPA-trastuzumab Fab-PEG_24_-EGF bsRICs or bsRICs modified with 2,2′,2″,2‴-(1,4,7,10-tetraazacyclododecane-1,4,7,10-tetrayl)tetraacetic acid (DOTA) to complex the β-particle emitter, ^177^Lu [t_1/2_ = 6.7 days; Eβmax = 0.50 MeV (78.6%), 0.38 MeV (9.1%), 0.18 MeV (12.2%)] administered at the No Observable Adverse Effect Level (NOAEL) inhibited the growth of s.c. MDA-MB-231/H2N tumors and trastuzumab-resistant TrR1 tumors that co-expressed HER2 and EGFR.^16^ These studies demonstrated that targeting HER2 and/or EGFR using bsRICs is a feasible approach for theranostic imaging and RIT of tumors that express these two receptors.

Positron emission tomography (PET) is a more sensitive imaging modality than SPECT. Thus, we constructed analogous bsRICs modified with 1,4,7-triazacyclononane-1,4,7-triacetic acid (NOTA) to complex the positron-emitter, ^64^Cu [t_1/2_ = 12.7 h; Eβ^+^ = 0.7 MeV (19%)] for the PET imaging of tumors that express HER2 and/or EGFR.^17^ These [^64^Cu]Cu-NOTA-trastuzumab Fab-PEG_24_-EGF bsRICs imaged s.c. HER2-negative/EGFR-positive MDA-MB-468, HER2-positive/EGFR-positive MDA-MB-231/H2N, or HER2-positive/EGFR-negative SK-OV-3 tumors in non-obese diabetic severe combined immunodeficiency (NOD-SCID) mice at 24 or 48 h p.i. An important finding was that the PEG_24_ linker prolonged the residence time in the blood compared to bsRICs without a PEG_24_ linker, which may contribute to higher tumor uptake of bsRICs compared to monospecific [^64^Cu]Cu-NOTA-trastuzumab Fab or [^64^Cu]Cu-NOTA-EGF. These studies were performed in NOD-SCID mice with s.c. tumors formed by inoculating tumor cells that homogeneously express HER2 and/or EGFR, but it would be useful to be able to engineer an experimental tumor xenograft model in mice composed of tumor cells that express HER2 or EGFR or have predefined receptor hetereogeneity by inoculating a mixed population of tumor cells expressing these two receptors. The ability to predefine tumor receptor homogeneity or heterogeneity would enable an investigation of these bsRICs for imaging or RIT under different conditions of receptor heterogeneity. We report here for the first time the establishment and characterization of tumor xenograft mouse models with predefined HER2 and/or EGFR expression, and demonstrate that [^64^Cu]Cu-NOTA-trastuzumab Fab-PEG_24_-EGF bsRICs were able to exploit receptor heterogeneity for tumor imaging by PET.

## Results

### Flow cytometry for human HER2-positive and EGFR-positive cells in populations of MDA-MB-468 and/or SK-OV-3 cells

**Figure 1:**
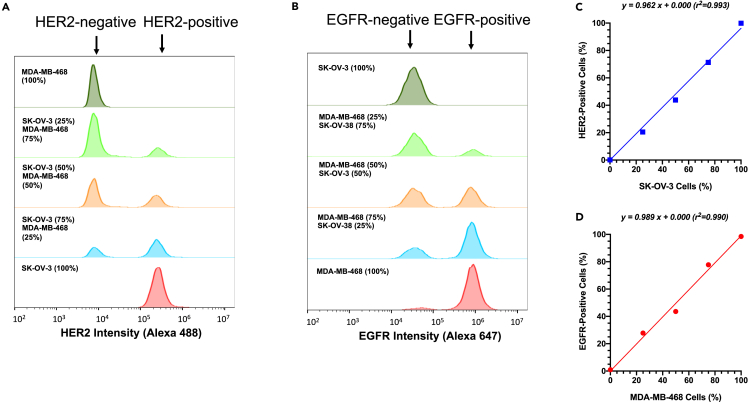
Flow cytometry for HER2-positive and EGFR-positive cells in populations of SK-OV-3 and/or MDA-MB-468 cells SK-OV-3 or MDA-MB-468 cells or mixtures of these cells immunostained with (A) anti-HER2 Alexa Fluor 488-conjugated trastuzumab or (B) with anti-EGFR Alexa Fluor 647-conjugated panitumumab were analyzed by flow cytometry. The proportion of HER2-positive or EGFR-positive cells was quantified as the area under the relevant peak for SK-OV-3 or MDA-MB-468 cells, respectively and plotted vs. the percentage of (C) SK-OV-3 cells or (D) MDA-MB-468 cells in the cell population.

Increasing the proportion of MDA-MB-468 human BC cells (EGFR-positive/HER2-negative) mixed with SK-OV-3 human ovarian cancer cells (HER2-positive/EGFR-negative) from 0% to 100% provided a cell population that was homogeneously HER2-positive, or exhibited an increasing gradient of EGFR-positive cells, or was homogeneously EGFR-positive. Similarly, increasing the proportion of SK-OV-3 cells mixed with MDA-MB-468 cells from 0% to 100%, provided a cell population that was homogeneously EGFR-positive, or exhibited an increasing gradient of HER2-positive cells, or was homogeneously HER2-positive. Flow cytometric analysis of cells immunostained with anti-HER2 Alexa Fluor 488-conjugated trastuzumab characterized cell populations containing 100% SK-OV-3 cells or mixtures of SK-OV-3 cells with 25%, 50%, 75%, or 100% MDA-MB-468 cells as 99.9%, 65.9%, 35.5%, 19.9% and 0% HER2-positive, respectively (Figure 1A). Similarly, flow cytometry of cells immunostained with anti-EGFR Alexa Fluor 647-conjugated panitumumab characterized cell populations containing 100% MDA-MB-468 cells or mixtures of MDA-MB-468 cells with 25%, 50%, 75% or 100% SK-OV-3 cells as 93.6%, 71.3%, 47.2, 20.2% and 0.3% EGFR-positive, respectively (Figure 1B). There was a strong linear correlation between the percent of SK-OV-3 and MDA-MB-468 cells and HER2 or EGFR positivity of cell populations, respectively (Figures 1C and 1D). These results validated the flow cytometric method for measuring the proportion of HER2-positive and EGFR-positive cells in tumor cell mixtures. This method was used to characterize the percent of HER2-positive and EGFR-positive cells *ex vivo* in s.c. tumor xenografts in NOD-SCID mice formed by the inoculation of 100% SK-OV-3 or MDA-MB-468 cells or a mixture of 30% SK-OV-3 cells and 70% MDA-MB-468 cells. Receptor characterization of these tumors was further used to interpret the results of PET imaging and biodistribution studies.

### Establishment of tumors homogeneous or heterogeneous for HER2 and EGFR in non-obese diabetic severe combined immunodeficiency mice

**Figure 2:**
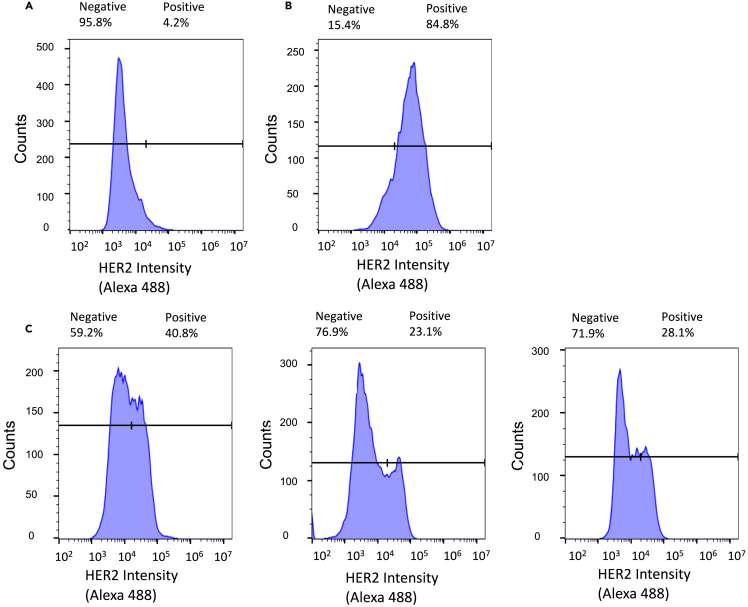
Flow cytometry for HER2-positive cells in dissociated tumors composed of SK-OV-3 and/or MDA-MB-468 cells Tumor xenografts in NOD/SCID mice were dissociated at 6 weeks post s.c. inoculation of tumor cells then the recovered cells were immunostained with anti-HER2 Alexa Fluor 488-conjugated trastuzumab and analyzed by flow cytometry. (A) Based on gating thresholds, MDA-MB-468 tumors were assigned as 4.2% HER2-positive cells and 95.8% HER2-negative cells. (B) SK-OV-3 tumors were assigned as 84.6% HER2-positive cells and 15.4% HER2-negative cells. (C) Replicate tumors (*n* = 3) formed by the inoculation of 30% SK-OV-3 cells and 70% MDA-MB-468 cells were assigned as 40.8%, 23.1%, and 28.1% HER2-positive cells and 59.2%, 76.9%, and 71.9% HER2-negative cells.

**Figure 3:**
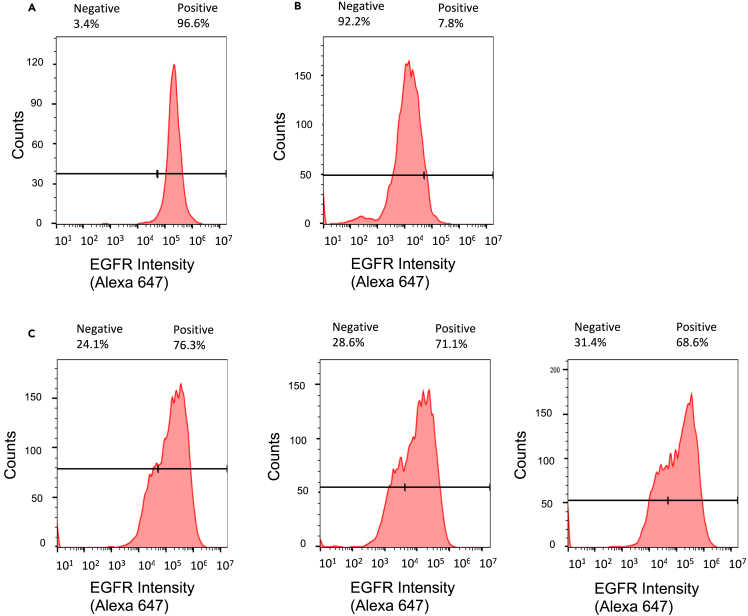
Flow cytometry for EGFR-positive cells in dissociated tumors composed of MDA-MB-468 and/or SK-OV-3 cells Tumor xenografts in NOD/SCID mice were dissociated at 6 weeks post s.c. inoculation of tumor cells then the recovered cells were immunostained with anti-EGFR Alexa Fluor 647-conjugated panitumumab and analyzed by flow cytometry. (A) Based on gating thresholds, MDA-MB-468 tumors were assigned as 96.6% EGFR-positive cells and 3.4% EGFR-negative cells. (B) SK-OV-3 tumors were assigned as 7.8% EGFR-positive cells and 92.2% EGFR-negative cells. (C) Replicate tumors (*n* = 3) formed by the inoculation of 70% MDA-MB-468 cells and 30% SK-OV-3 cells were assigned as 76.3%, 71.4%, and 68.6% EGFR-positive cells and 24.1%, 28.6%, and 31.4% EGFR-negative cells.

The HER2 and EGFR expression of tumor xenografts in NOD-SCID mice established by the s.c. inoculation of SK-OV-3 or MDA-MB-468 cells or a mixture of SK-OV-3 cells (30%) and MDA-MB-468 cells (70%) at 6 weeks post-inoculation were assessed by flow cytometry of the dissociated tumor cells. The percentage of SK-OV-3 and MDA-MB-468 cells inoculated to form tumors with heterogeneous receptor expression was selected based on the relative doubling times of these cells *in vitro* (19.2 h and 33.5 h, respectively). The gating thresholds for HER2-positive or EGFR-positive cells were set by analyzing SK-OV-3 or MDA-MB-468 cells, respectively, or mixtures of these cells at known proportions (Figure 1). Based on these gating thresholds, cells dissociated from tumors formed of MDA-MB-468 cells immunostained with anti-HER2 Alexa Fluor 488-conjugated trastuzumab were assigned as 95.8% HER2-negative and 4.2% HER2-positive (Figure 2A). Cells dissociated from tumors formed of SK-OV-3 cells were assigned as 84.6% HER2-positive and 15.4% HER2-negative (Figure 2B). Analysis of dissociated replicate tumors (*n* = 3) formed by the inoculation of a mixture of 30% SK-OV-3 cells and 70% MDA-MB-468 cells were assigned as 40.8%, 23.1% and 28.1% HER2-positive cells (mean ± SD = 30.7 ± 7.4%) and 59.2%, 76.9% and 71.9% HER2-negative cells (mean ± SD = 69.3 ± 7.4%; Figure 2C). Cells dissociated from tumors at 6 weeks post-inoculation of MDA-MB-468 cells in NOD/SCID mice, immunostained with anti-EGFR Alexa Fluor 647-conjugated panitumumab and analyzed by flow cytometry were assigned based on gating thresholds as 96.6% EGFR-positive cells and 3.4% EGFR-negative cells (Figure 3A). SK-OV-3 tumors were assigned as 92.2% EGFR-negative cells and 7.8% EGFR-positive cells (Figure 3B). Replicate tumors (*n* = 3) formed by the inoculation of 70% MDA-MB-468 cells and 30% SK-OV-3 cells were assigned as 76.3%, 71.4% and 68.6% EGFR-positive cells (mean ± SD = 72.1 ± 3.2%) and 24.1%, 28.6% and 31.4% EGFR-negative cells (mean ± SD = 28.0 ± 3.0%; Figure 3C).

**Figure 4:**
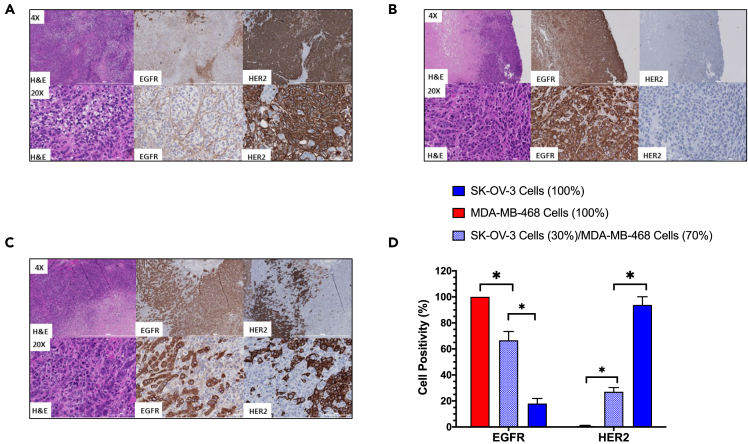
Immunohistochemical (IHC) staining of tumors composed of SK-OV-3 and/or MDA-MB-468 cells for HER2-positive and EGFR-positive cells Representative sections of tumors formed by the inoculation of (A) SK-OV-3 cells or (B) MDA-MB-468 cells or (C) a mixture of 30% SK-OV-3 cells and 70% MDA-MB-468 cells at 6 weeks post-inoculation in NOD/SCID mice. Sections were stained with hematoxylin and eosin (H&E) or immunostained for HER2 or EGFR. Sections are shown at 4× or 20× amplification. (D) The percentage of cells immunopositive for HER2 or EGFR in replicate tumor sections was quantified by image analysis and is shown for SK-OV-3 or MDA-MB-468 tumors or tumors formed by inoculating a mixture of 30% SK-OV-3 cells and 70% MDA-MB-468 cells. Data shown are mean ± SD (*n* = 3). Statistically significant differences (Student’s t test; *p* < 0.05) are indicated by the asterisks.

We assessed the spatial distribution of HER2 and EGFR expression in tumors formed by the s.c. inoculation of SK-OV-3 or MDA-MB-468 cells or a mixture of 30% SK-OV-3 and 70% MDA-MB-468 cells in NOD/SCID mice by immunohistochemical (IHC) staining. SK-OV-3 tumors exhibited strong and homogeneous immunopositivity for HER2 (Figure 4A), while MDA-MB-468 tumors exhibited strong and homogeneous immunopositivity for EGFR (Figure 4B). Replicate tumors (*n* = 3) formed by the inoculation of a mixture of 30% SK-OV-3 cells and 70% MDA-MB-468 cells exhibited spatially complementary HER2 or EGFR immunostaining (Figure 4C). The percentage of cells that were HER2-positive/HER2-negative or EGFR-positive/EGFR-negative in these sections was quantified by image analysis (Figure 4D). MDA-MB-468 tumors contained 100 ± 0% EGFR-positive cells and 0.7 ± 0.1% HER2-positive cells. SK-OV-3 tumors contained 94 ± 6% HER2-positive cells and 18 ± 4% EGFR-positive cells. Some cells stained positive for HER2 and EGFR, resulting in >100% cell staining for these receptors. In tumors formed by inoculating mixtures of SK-OV-3 and MDA-MB-468 cells, there were 27 ± 3% HER2-positive cells and 67 ± 7% EGFR-positive cells (Figure 4D) which were significantly different (*p* < 0.05) than tumors composed only of SK-OV-3 or MDA-MB-468 cells. The proportion of HER2-positive and EGFR-positive cells in tumors measured *ex vivo* by flow cytometry or by IHC staining agreed with the percentage of SK-OV-3 and MDA-MB-468 cells inoculated in NOD-SCID mice to form these tumors.

### Positron emission tomography/computed tomography imaging and biodistribution studies reveal that bispecific radioimmunoconjugates exploit receptor heterogeneity

**Figure 5:**
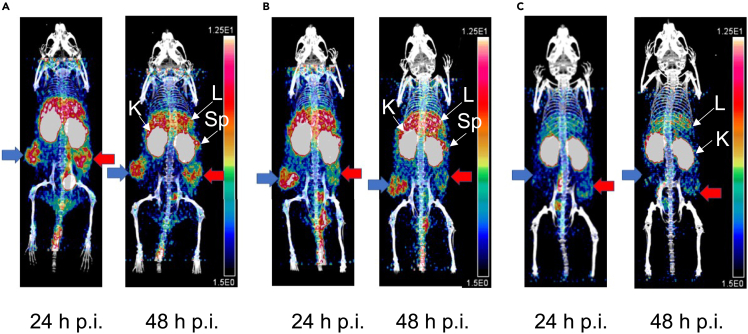
PET/CT images of NOD-SCID mice with s.c. SK-OV-3 tumors with homogeneous HER2 expression or tumors with heterogeneous HER2 expression formed from a mixture of SK-OV-3 and MDA-MB-468 cells Representative images (supine position) of NOD-SCID mice with s.c. SK-OV-3 tumors on the right flank that homogeneously express HER2 (blue arrow) and tumors formed by the inoculation of a mixture of SK-OV-3 cells (30%) and MDA-MB-468 cells (70%) with heterogeneous HER2 and EGFR expression on the left flank (red arrow). Images were obtained at 24 h and 48 h p.i. of (A) [^64^Cu]Cu-NOTA-trastuzumab Fab-PEG_24_-EGF, (B) [^64^Cu]Cu-NOTA-trastuzumab Fab or (C) [^64^Cu]Cu-NOTA-EGF. All images were adjusted to the same intensity and the intensity scale (ranging from 1.5% ID/g to 12.5% ID/g) is shown at the right of each set of images. Normal organ uptake is visible on the 24 h and 48 h p.i. images but is labeled on the 48 h p.i. images. L: liver; K: kidneys; Sp: spleen.

PET/CT images (supine position) were obtained at 24 or 48 h p.i. of [^64^Cu]Cu-NOTA-trastuzumab Fab-PEG_24_-EGF bsRICs or monospecific [^64^Cu]Cu-NOTA-trastuzumab Fab or [^64^Cu]Cu-NOTA-EGF in NOD-SCID mice with s.c. SK-OV-3 tumors or tumors formed by inoculating mixtures of SK-OV-3 (30%) and MDA-MB-468 cells (70%). SK-OV-3 tumors homogeneously expressing HER2 (right flank) and tumors formed from mixtures of SK-OV-3 and MDA-MB-468 cells heterogeneously expressing HER2 and EGFR (left flank) were imaged at 24 h and 48 h p.i. of [^64^Cu]Cu-NOTA-trastuzumab Fab-PEG_24_-EGF bsRICs (Figure 5A). Monospecific [^64^Cu]Cu-NOTA-trastuzumab Fab most strongly imaged SK-OV-3 tumors (right flank; Figure 5B) but there was much lower signal intensity for tumors with heterogeneous HER2 and EGFR expression (left flank; Figure 5B). Monospecific [^64^Cu]Cu-NOTA-EGF identified tumors that were heterogeneous for HER2 and EGFR (left flank; Figure 5C) but not SK-OV-3 tumors that homogeneously expressed HER2 (right flank; Figure 5C). Normal organ uptake was observed in the liver and kidneys for both bsRICs and monospecific agents but also in the spleen for bsRICs and [^64^Cu]Cu-NOTA-trastuzumab Fab. Spleen uptake was not visualized for [^64^Cu]Cu-NOTA-EGF.

**Figure 6:**
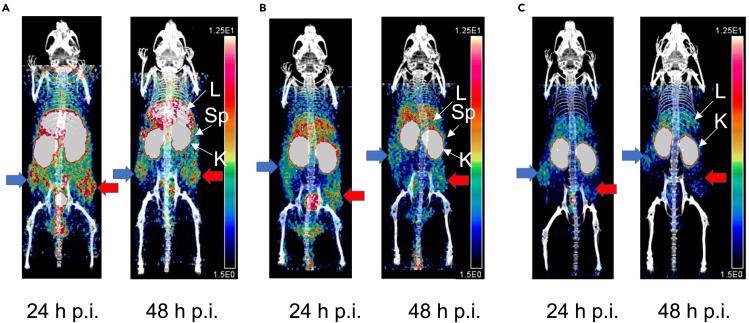
PET/CT images of NOD-SCID mice with s.c. MDA-MB-468 tumors with homogeneous EGFR expression or tumors with heterogeneous EGFR expression formed from a mixture of MDA-MB-468 and SK-OV-3 cells Representative images (supine position) of NOD-SCID mice with s.c. MDA-MB-468 tumors on the right flank that homogeneously express EGFR (blue arrow) and tumors formed by the inoculation of a mixture of MDA-MB-468 cells (70%) and SK-OV-3 cells (30%) with heterogeneous HER2 and EGFR expression on the left flank (red arrow). Images were obtained at 24 h and 48 h p.i. of (A) [^64^Cu]Cu-NOTA-trastuzumab Fab-PEG_24_-EGF, (B) [^64^Cu]Cu-NOTA-trastuzumab Fab or (C) [^64^Cu]Cu-NOTA-EGF. All images were adjusted to the same intensity and the intensity scale (ranging from 1.5% ID/g to 12.5% ID/g) is shown at the right of each set of images. Normal organ uptake is visible on the 24 h and 48 h p.i. images but is labeled on the 48 h p.i. images. L: liver; K: kidneys; Sp: spleen.

PET/CT images (supine position) were obtained at 24 h or 48 h p.i. of [^64^Cu]Cu-NOTA-trastuzumab Fab-PEG_24_-EGF bsRICs or [^64^Cu]Cu-NOTA-trastuzumab Fab or [^64^Cu]Cu-NOTA-EGF in NOD-SCID mice with s.c. MDA-MB-468 tumors that homogeneously express EGFR or tumors formed from mixtures of SK-OV-3 cells (30%) and MDA-MB-468 cells (70%) with heterogeneous HER2 and EGFR expression. MDA-MB-468 tumors (right flank) and tumors heterogeneous for EGFR and HER2 (left flank) were imaged at 24 h or 48 h p.i. of [^64^Cu]Cu-NOTA-trastuzumab Fab-PEG_24_-EGF bsRICs (Figure 6A). Monospecific [^64^Cu]Cu-NOTA-trastuzumab Fab showed MDA-MB-468 tumors (right flank) and tumors that were heterogeneous for EGFR and HER2 (left flank) but the signal intensity appeared slightly higher in tumors that co-expressed EGFR and HER2 (Figure 6B). [^64^Cu]Cu-NOTA-EGF imaged MDA-MB-468 tumors (right flank) but not tumors that were heterogeneous for EGFR and HER2 (left flank; Figure 6C).

The tumor and normal tissue uptake of [^64^Cu]Cu-NOTA-trastuzumab Fab-PEG_24_-EGF bsRICs or monospecific [^64^Cu]Cu-NOTA-trastuzumab Fab or [^64^Cu]Cu-NOTA-EGF were determined at 48 h p.i. in mice with SK-OV-3 tumors or tumors formed from a mixture of SK-OV-3 cells (30%) and MDA-MB-468 cells (70%). Tumor uptake of [^64^Cu]Cu-NOTA-trastuzumab Fab-PEG_24_-EGF bsRICs was 1.7-fold higher in SK-OV-3 tumors homogeneously expressing HER2 (8.2 ± 1.7% ID/g) than in tumors with heterogeneous HER2 and EGFR expression but this difference was not significant (4.8 ± 0.6% ID/g; *p* > 0.05; Table 1). There were no significant differences (*p* > 0.05) in the uptake of [^64^Cu]Cu-NOTA-trastuzumab Fab (6.3 ± 0.2% ID/g) and [^64^Cu]Cu-NOTA-trastuzumab Fab-PEG_24_-EGF (8.2 ± 1.7% ID/g) in SK-OV-3 tumors. However, uptake of bispecific [^64^Cu]Cu-NOTA-trastuzumab Fab-PEG_24_-EGF (4.8 ± 0.6% ID/g) in tumors with heterogeneous HER2 and EGFR expression was 1.5-fold significantly greater than monospecific [^64^Cu]Cu-NOTA-trastuzumab Fab (3.3 ± 0.3% ID/g) and 2.5-fold significantly higher than [^64^Cu]Cu-NOTA-EGF (1.9 ± 0.4% ID/g; *p* < 0.05; Table 1). Furthermore, monospecific [^64^Cu]Cu-NOTA-EGF exhibited 5.1-fold significantly lower uptake in SK-OV-3 tumors (1.6 ± 0.3% ID/g; *p* < 0.05) than bispecific [^64^Cu]Cu-NOTA-trastuzumab Fab-PEG_24_-EGF and 3.9-fold significantly lower uptake than monospecific [^64^Cu]Cu-NOTA-trastuzumab Fab. [^64^Cu]Cu-NOTA-EGF exhibited 2.5-fold significantly lower uptake than bispecific [^64^Cu]Cu-NOTA-trastuzumab Fab-PEG_24_-EGF and 1.7-fold significantly lower uptake than monospecific [^64^Cu]Cu-NOTA-trastuzumab Fab in tumors with heterogeneous HER2 and EGFR expression (*p* < 0.05; Table 1). Lower tumor uptake of [^64^Cu]Cu-NOTA-EGF compared to other agents may be due to its more rapid elimination from the blood, since blood activity at 48 h p.i. (0.4 ± 0.1% ID/g; *p* < 0.05) was significantly lower than [^64^Cu]Cu-NOTA-trastuzumab Fab-PEG_24_-EGF bsRICs (1.3 ± 0.2% ID/g) or [^64^Cu]Cu-NOTA-trastuzumab Fab (1.2 ± 0.2% ID/g), whereas there were no significant differences in blood activity between [^64^Cu]Cu-NOTA-trastuzumab Fab-PEG_24_-EGF and [^64^Cu]Cu-NOTA-trastuzumab Fab. Normal tissue uptake was greatest for all agents in the liver, kidneys and spleen, but spleen uptake was 20.8-fold lower for [^64^Cu]Cu-NOTA-EGF (0.9 ± 0.1% ID/g) than [^64^Cu]Cu-NOTA-trastuzumab Fab-PEG_24_-EGF (18.7 ± 4.3% ID/g) and 12.6-fold lower than [^64^Cu]Cu-NOTA-trastuzumab Fab (11.3 ± 1.6% ID/g; *p* < 0.05; Table 1).

**Table 1:** Tumor and normal tissue biodistribution at 48 h p.i. in NOD-SCID mice with SK-OV-3 tumors and tumors formed from mixtures of SK-OV-3 and MDA-MB-468 cells

Tissue	Percent injected dose/g (% ID/g)^a^		
	[^64^Cu]Cu-NOTA-trastuzumab Fab-PEG_24_-EGF	[^64^Cu]Cu-NOTA-trastuzumab Fab	[^64^Cu]Cu-NOTA-EGF
Blood	1.3 ± 0.2	1.2 ± 0.2	0.4 ± 0.1^b^^,^^c^
Heart	1.3 ± 0.1	1.5 ± 0.1	0.4 ± 0.06
Lungs	1.8 ± 0.2	4.0 ± 2.5	0.8 ± 0.1
Liver	7.8 ± 1.4	5.0 ± 2.1	2.4 ± 0.1
Kidneys	52.1 ± 10.4	43.5 ± 8.3	28.0 ± 11.8
Spleen	18.7 ± 4.3	11.3 ± 1.6	0.9 ± 0.1^b^^,^^c^
Stomach	1.0 ± 0.2	0.5 ± 0.1	0.3 ± 0.1
Intestine	1.7 ± 0.3	1.9 ± 0.2	0.9 ± 0.1
Muscle	0.6 ± 0.3	0.6 ± 0.1	0.2 ± 0.1
Skin	0.8 ± 0.1	0.8 ± 0.03	0.4 ± 0.04
Tail	4.4 ± 1.6	4.3 ± 1.8	1.2 ± 0.7
SK-OV-3 (100%)	8.2 ± 1.7	6.3 ± 0.2	1.6 ± 0.3^b^^,^^c^
SK-OV-3 (30%)/MDA-MB-468 (70%)	4.8 ± 0.6^c^	3.3 ± 0.3^b^	1.9 ± 0.4^b^^,^^c^

a Values shown are the mean ± SEM (n = 4–6).

b Significantly different compared to bsRICs (*p* < 0.05).

c Significantly different compared to [^64^Cu]Cu-NOTA-trastuzumab Fab (*p* < 0.05).

The tumor and normal tissue uptake of bispecific [^64^Cu]Cu-NOTA-trastuzumab Fab-PEG_24_-EGF or monospecific [^64^Cu]Cu-NOTA-trastuzumab Fab or [^64^Cu]Cu-NOTA-EGF were determined at 48 h p.i. in mice with MDA-MB-468 tumors or tumors formed by inoculating a mixture of MDA-MB-468 cells (70%) and SK-OV-3 cells (30%). The uptake of [^64^Cu]Cu-NOTA-trastuzumab Fab-PEG_24_-EGF in MDA-MB-468 tumors (3.9 ± 0.3% ID/g) was 1.6-fold lower than in tumors with heterogeneous HER2 and EGFR expression (6.2 ± 1.2% ID/g) but this difference was not statistically significant (*p* > 0.05; Table 2). The uptake of monospecific [^64^Cu]Cu-NOTA-trastuzumab Fab and [^64^Cu]Cu-NOTA-EGF in MDA-MB-468 tumors were 1.9-fold and 1.6-fold significantly lower, respectively, than bispecific [^64^Cu]Cu-NOTA-trastuzumab Fab-PEG_24_-EGF (2.1 ± 0.5% ID/g and 2.4 ± 0.5% ID/g, respectively; *p* < 0.05; Table 2). In tumors with heterogeneous HER2 and EGFR expression, the uptake of monospecific [^64^Cu]Cu-NOTA-trastuzumab Fab or [^64^Cu]Cu-NOTA-EGF was 1.9-fold and 3.0-fold significantly lower, respectively, than bispecific [^64^Cu]Cu-NOTA-trastuzumab Fab-PEG_24_-EGF (3.3 ± 0.6% ID/g and 2.1 ± 0.2% ID/g vs. 6.2 ± 1.2% ID/g, respectively; *p* < 0.05; Table 2). There were no significant differences in the uptake of [^64^Cu]Cu-NOTA-trastuzumab Fab or [^64^Cu]Cu-NOTA-EGF in MDA-MB-468 tumors or in tumors with heterogeneous HER2 and EGFR expression. Blood activity at 48 h p.i. was 2.0-fold higher for [^64^Cu]Cu-NOTA-trastuzumab Fab-PEG_24_-EGF (2.0 ± 0.4% ID/g) than [^64^Cu]Cu-NOTA-trastuzumab Fab (1.0 ± 0.3% ID/g) but this difference was not significant (*p* > 0.05). However, blood activity was 5-fold significantly lower for [^64^Cu]Cu-NOTA-EGF (0.4 ± 0.1% ID/g; *p* < 0.05; Table 2). Normal tissue uptake was highest for all agents in the liver, kidneys and spleen, but spleen uptake was 9.7-fold lower for [^64^Cu]Cu-NOTA-EGF (1.1 ± 0.1% ID/g) than [^64^Cu]Cu-NOTA-trastuzumab Fab-PEG_24_-EGF (10.7 ± 1.4% ID/g) and 12.4-fold lower than [^64^Cu]Cu-NOTA-trastuzumab Fab (13.6 ± 2.0% ID/g; *p* < 0.05; Table 2). Uptake in the kidneys for [^64^Cu]Cu-NOTA-EGF (29.9 ± 2.8% ID/g) was 2.1-fold lower than [^64^Cu]Cu-NOTA-trastuzumab Fab (62.6 ± 11.1% ID/g; *p* < 0.05).

**Table 2:** Tumor and normal tissue biodistribution at 48 h p.i. in NOD-SCID mice with MDA-MB-468 tumors and tumors formed from mixtures of MDA-MB-468 and SK-OV-3 cells

Tissue	Percent injected dose/g (% ID/g)^a^		
	[^64^Cu]Cu-NOTA-trastuzumab Fab-PEG_24_-EGF	[^64^Cu]Cu-NOTA-trastuzumab Fab	[^64^Cu]Cu-NOTA-EGF
Blood	2.0 ± 0.4	1.0 ± 0.3	0.4 ± 0.1 b
Heart	1.9 ± 0.2	1.2 ± 0.3	0.5 ± 0.02
Lungs	4.1 ± 2.2	2.2 ± 0.8	1.0 ± 0.1
Liver	6.9 ± 3.0	5.5 ± 1.0	2.5 ± 0.2
Kidneys	38.1 ± 6.1	62.6 ± 11.1	29.9 ± 2.8^c^
Spleen	10.7 ± 1.4	13.6 ± 2.0	1.1 ± 0.1^b^^,^^c^
Stomach	0.6 ± 0.2	0.7 ± 0.3	0.3 ± 0.1
Intestine	2.1 ± 0.2	1.6 ± 0.3	1.0 ± 0.1
Muscle	0.6 ± 0.1	0.6 ± 0.4	0.2 ± 0.04
Skin	0.8 ± 0.1	0.7 ± 0.3	0.6 ± 0.2
Tail	3.7 ± 0.3	3.7 ± 0.7	0.9 ± 0.2
MDA-MB-468 (100%)	3.9 ± 0.3	2.1 ± 0.5^b^	2.4 ± 0.5^b^
SK-OV-3 (30%)/MDA-MB-468 (70%)	6.2 ± 1.2	3.3 ± 0.6^b^	2.1 ± 0.2^b^

a Values shown are the mean ± SEM (n = 4–6).

b Significantly different compared to bsRICs (*p* < 0.05).

c Significantly different compared to [^64^Cu]Cu-NOTA-trastuzumab Fab (*p* < 0.05).

## Discussion

Receptor heterogeneity is a challenge for targeted cancer therapies since it may lead to poor response to treatment and moreover, may apply evolutionary pressure to select tumor cells that do not express the target.^18^ One approach to overcome receptor heterogeneity may be to use agents that recognize more than one target. Our previous studies showed that SPECT with [^111^In]In-DTPA-trastuzumab Fab-PEG_24_-EGF^15^ and PET with [^64^Cu]Cu-NOTA-trastuzumab Fab-PEG_24_-EGF^17^ bsRICs imaged tumors in mice that express HER2 or EGFR or coexpress these receptors. We further found that the growth of tumors expressing HER2, EGFR or both receptors in mice was inhibited by RIT with Auger electron-emitting [^111^In]In-DTPA-trastuzumab Fab-PEG_24_-EGF or β-particle emitting [^177^Lu]Lu-DOTA-trastuzumab Fab-PEG_24_-EGF.^16^ However, these studies did not examine the ability of bsRICs to localize in tumors with receptor heterogeneity arising from mixed populations of tumor cells that express HER2 or EGFR. We describe here for the first time tumor xenografts in NOD-SCID mice with predefined receptor heterogeneity formed by the s.c. inoculation of mixtures of HER2-positive/EGFR-negative SK-OV-3 human ovarian cancer cells and EGFR-positive/HER2-negative MDA-MB-468 human BC cells. The tumor localization of [^64^Cu]Cu-NOTA-trastuzumab Fab-PEG_24_-EGF bsRICs in these tumors with heterogeneous receptor expression was evaluated by PET and biodistribution studies and compared to tumors formed of SK-OV-3 cells or MDA-MB-468 cells that homogeneously express HER2 or EGFR, respectively. In addition, the tumor localization of these bsRICs was compared to monospecific [^64^Cu]Cu-NOTA-trastuzumab Fab or [^64^Cu]Cu-NOTA-EGF. Only one other study has reported tumors in mice that heterogeneously express HER2 formed by the inoculation of mixtures of HER2-positive and HER2-negative tumor cells.^19^ In that study, tumors composed of HER2-positive TUBO murine mammary carcinoma tumors or a HER2-negative subclone, TUBO-P2J or mixtures of these cells were inoculated s.c. in Balb/c mice. Tumors formed from mixtures of these two cell types exhibited HER2 heterogeneity and did not respond to treatment with anti-neu antibodies (analogous to anti-HER2 trastuzumab). However, that study did not evaluate bispecific antibodies that recognize other targets coexpressed with HER2.

SK-OV-3 human ovarian cancer cells and MDA-MB-468 human BC cells were chosen to establish tumors that homogeneously or heterogeneously expressed HER2 and EGFR since SK-OV-3 cells are HER2-positive but have negligible EGFR expression^20^ while MDA-MB-468 cells are EGFR-positive but have negligible HER2^21^ (Figure 1). Both cell types are tumorigenic in mice. We did not select SK-BR-3 or BT-474 human BC cells as a HER2-positive tumor cell type because SK-BR-3 cells are poorly tumorigenic^22^ and SK-BR-3 and BT-474 cells coexpress EGFR at moderate or low levels, respectively.^23^ Our intent was to establish tumors that heterogeneously expressed HER2 and EGFR on different tumor cell populations. Flow cytometry accurately measured the percentage of HER2-positive SK-OV-3 and EGFR-positive MDA-MB-468 cells in mixtures of these two cell types (Figures 2 and 3). It was important to select the proportion of SK-OV-3 and MDA-MB-468 cells in cell mixtures for s.c. inoculation into NOD-SCID mice to establish tumors with receptor heterogeneity, since there is a risk that one cell type may out-compete the other as tumors grow due to differences in cell doubling times *in vivo*. Therefore, we first measured the cell doubling times *in vitro*, which were 19.2 h and 33.5 h for SK-OV-3 and MDA-MB-468 cells, respectively. To avoid overgrowth of SK-OV-3 cells, we inoculated a mixture of 30% SK-OV-3 and 70% MDA-MB-468 cells s.c. in NOD-SCID mice to form tumors with heterogeneous HER2 and EGFR expression. However, analysis of the percentage of HER2-positive and EGFR-positive cells from replicate tumors (*n* = 3) at 6 weeks post-inoculation *ex vivo* by flow cytometry revealed 30.7 ± 7.4% of cells assigned as HER2-positive (Figure 2C) and 72.1 ± 3.2%) EGFR-positive cells (Figure 3C), which was almost identical to the proportion of SK-OV-3 and MDA-MB-468 cells inoculated, respectively. These results were not due to an artifact caused by isolating tumor cells *ex vivo*, since image analysis of tumor sections immunostained for HER2 and EGFR similarly revealed 27 ± 3% HER2-positive cells and 67 ± 7% EGFR-positive cells (Figures 4C and 4D). Thus, it appears that *in vivo*, the cell doubling times of SK-OV-3 and MDA-MB-468 cells were similar. There were spatially complementary HER2-positive and EGFR-positive regions in these tumors (Figure 4C). In contrast, tumors formed only from SK-OV-3 or MDA-MB-468 cells exhibited homogeneous HER2-positive/EGFR-negative or EGFR-positive/HER2-negative cells, respectively by flow cytometry (Figures 2A, 2B, 3A, and 3B) and the corresponding homogeneous HER2 or EGFR immunostaining (Figures 4A and 4B).

Using *in situ* hybridization (ISH) techniques to identify *HER2* gene amplifications, three distinct patterns of HER2 heterogeneity in human BC tumors have been described: i) “clustered type” with regions of *HER2* amplified and non-*HER2* amplified cells, ii) “mosaic type” with interspersed *HER2* amplified and non-*HER2* amplified cells, and iii) “scattered type” with isolated *HER2*-amplified cells in a tumor composed mainly of non-*HER2* amplified cells.^24^ By combining ISH and IHC staining, two patterns of HER2 heterogeneity were defined: i) “genetic heterogeneity” consisting of a mixture of HER2-positive and HER2-negative cells due to corresponding *HER2-*amplification or non-*HER2* gene amplification, and ii) “non-genetic heterogeneity” consisting of a mixture of HER2-positive and HER2-negative cells by ICH despite homogeneous *HER2* gene amplification.^24^ SK-OV-3 cells are *HER2* gene-amplified^25^ while MDA-MB-468 cells are not amplified for the *HER2* gene, thus the pattern of HER2 heterogeneity in tumors formed of a mixture of SK-OV-3 and MDA-MB-468 cells in our study could be classified as “genetic heterogeneity.” It should be noted that in patients with HER2-positive BC, besides intratumoral HER2 heterogeneity, there may be interlesional HER2 heterogeneity. The ZEPHIR trial investigated PET imaging with [^89^Zr]Zr-DFO-trastuzumab to predict response to treatment with trastuzumab emtansine (T-DM1) in patients with HER2-positive BC.^26^ PET revealed no tumor uptake of [^89^Zr]Zr-DFO-trastuzumab in 29% of patients and heterogeneous interlesional uptake in 46% of patients. Combining PET with [^89^Zr]Zr-DFO-trastuzumab and [^18^F]F-2-fluorodeoxyglucose predicted response to T-DM1 and patient survival. In our study, we established two tumors in NOD-SCID mice – one on the right flank that was homogeneously HER2-positive or EGFR-positive and one on the left flank that was heterogeneous for the expression of HER2 and EGFR. We imaged mice with these tumors by PET with bispecific [^64^Cu]Cu-NOTA-trastuzumab Fab-PEG_24_-EGF or monospecific [^64^Cu]Cu-NOTA-trastuzumab Fab or [^64^Cu]Cu-NOTA-EGF (Figures 5 and 6). Imaging of tumors in this model that homogeneously express HER2 or are EGFR-positive but HER2-negative may simulate the scenario of interlesional HER2 heterogeneity in patients with HER2-positive BC. Imaging of mice with tumors that heterogeneously express HER2 and EGFR may simulate intralesional HER2 heterogeneity in patients with HER2-positive BC.

PET with [^64^Cu]Cu-NOTA-trastuzumab Fab-PEG_24_-EGF bsRICs imaged HER2-positive SK-OV-3 tumors and tumors with heterogeneous receptor expression formed by the inoculation of a mixture of SK-OV-3 and EGFR-positive MDA-MB-468 cells (Figure 5A) while monospecific [^64^Cu]Cu-NOTA-trastuzumab Fab imaged SK-OV-3 tumors but exhibited lower signal intensity in tumors with heterogeneous receptor expression (Figure 5B). PET with [^64^Cu]Cu-NOTA-EGF was unable to image SK-OV-3 tumors and exhibited a low signal for tumors with heterogeneous receptor expression (Figure 5C). Similarly, [^64^Cu]Cu-NOTA-trastuzumab Fab-PEG_24_-EGF bsRICs imaged EGFR-positive MDA-MB-468 tumors and tumors with heterogeneous receptor expression formed from a mixture of MDA-MB-468 and HER2-positive SK-OV-3 cells (Figure 6A) while monospecific [^64^Cu]Cu-NOTA-EGF only imaged MDA-MB-468 tumors (Figure 6C). [^64^Cu]Cu-NOTA-trastuzumab Fab imaged tumors with heterogeneous EGFR and HER2 expression but also MDA-MB-468 tumors with lower intensity (Figure 6B). Uptake of [^64^Cu]Cu-NOTA-trastuzumab Fab in HER2-negative MDA-MB-468 tumors may be explained by the enhanced permeability and retention (EPR) effect which mediates the non-specific tumor localization of macromolecules.^27^ Biodistribution studies (Tables 1 and 2) confirmed the PET imaging results comparing bsRICs with monospecific agents in tumors that homogeneously or heterogeneously expressed HER2 or EGFR.

In conclusion, bispecific [^64^Cu]Cu-NOTA-trastuzumab Fab-PEG_24_-EGF were able to exploit receptor heterogeneity and image tumors in NOD-SCID mice that homogeneously or heterogeneously expressed HER2 or EGFR, while monospecific [^64^Cu]Cu-NOTA-trastuzumab Fab or [^64^Cu]Cu-NOTA-EGF were limited to imaging tumors that expressed HER2 or EGFR. We previously reported that these bsRICs labeled with Auger electron-emitting, ^111^In or β-particle emitting ^177^Lu were effective for RIT of tumors that expressed HER2 or EGFR or co-expressed these two receptors.^16^ PET with [^64^Cu]Cu-NOTA-trastuzumab Fab-PEG_24_-EGF bsRICs may identify patients who benefit from RIT with ^111^In or ^177^Lu-labeled bsRIC that target HER2 and EGFR, enabling a theranostic approach. There was higher kidney uptake of [^64^Cu]Cu-NOTA-trastuzumab Fab-PEG_24_-EGF (Tables 1 and 2) than we previously reported for [^177^Lu]-DOTA-trastuzumab Fab-PEG_24_-EGF (∼8% ID/g)^16^ or [^111^In]In-DTPA-trastuzumab Fab-PEG_24_-EGF (5.8–6.5% ID/g).^15^ The reason is not known. However, we envision treatment with ^177^Lu- or ^111^In-labeled bsRICs which were non-toxic to the kidneys in mice.^16^ In the future, it may be possible to decrease the kidney uptake of ^64^Cu-labeled bsRICs by conjugating trastuzumab Fab through a PEG_24_ linker to anti-EGFR panitumumab (Vectibix, Amgen) Fab instead of EGF. This would increase the molecular weight of the bsRICs to >100 kDa, which exceeds the renal threshold for the filtration of proteins.^28^ Finally, our results illustrate that tumor xenografts with predefined heterogeneous receptor expression may be established by inoculating mixtures of tumor cells that express distinct receptors. This approach to establishing tumor xenografts in mice could be very useful to examine the effects of receptor heterogeneity on imaging and treating tumors with multi-targeted theranostic agents.

### Limitations of the study

The tumor xenograft models used in this study were composed of human SK-OV-3 ovarian cancer or MDA-MB-468 breast cancer cells which homogeneously and highly express HER2 or EGFR, respectively. The levels of HER2 and EGFR expression by these cells may be greater than found in human tumors. In addition, tumors with homogeneous or heterogeneous HER2 and EGFR expression were formed by inoculating these two cell types or mixtures of these cells. These tumors are experimental models used to compare the imaging properties of bispecific with monospecific agents under conditions of homogeneous or heterogeneous receptor expression but these conditions may not reflect the levels of these receptors or the receptor heterogeneity found in tumors in patients with HER2-positive BC. The tumor xenograft model exhibiting heterogeneous HER2 expression was not intended to incorporate resistance to HER2-targeted therapies, only receptor heterogeneity. Mediastinal activity and liver uptake of the bsRICs may interfere with the imaging of primary breast tumors or metastases of BC to the liver in patients.

## STAR★Methods

### Key resources table

**Table:** 

REAGENT or RESOURCE	SOURCE	IDENTIFIER
**Antibodies**		

Panitumumab	Amgen	Vectibix®; RRID: AB_2910958
Trastuzumab	Roche	Herceptin®; RRID: AB_3074204
[^64^Cu]Cu-NOTA-trastuzumab Fab-PEG_24_-EGF	Kwon L et al.^19^	NA
[^64^Cu]Cu-NOTA-trastuzumab Fab	Kwon L et al.^19^	NA
Rabbit anti-human EGFR monoclonal antibody	Cell Signaling Technology	#4267
Rabbit anti-human HER2 monoclonal antibody	ThermoFisher/Invitrogen	#SP3

**Chemicals, peptides, and recombinant proteins**		

Alexa Fluor™ 647 N-hydroxysuccinimide ester	ThermoFisher	A20006
Alexa Fluor™ 488 N-hydroxysuccinimide ester	ThermoFisher	A20000
[^64^Cu]Cu-NOTA-EGF	Kwon L et al.^19^	NA
Collagenase	Sigma-Aldrich	SCR103

**Experimental models: Cell lines**		

Human: SK-OV-3 cells	ATCC	SK-OV-3; Cat#HTB-77
Human: MDA-MB-468 cells	ATCC	MDA-MB-468; Cat#HTB-132

**Experimental models: Organisms/strains**		

Mouse: NOD-SCID	Princess Margaret Cancer Center, Toronto, ON, Canada	NOD.CB17*-Prkdc*^*scid*^/NCrCrl
Human: Tumor xenografts homogeneously or heterogeneously expressing HER2 and/or EGFR	This paper.	Inoculation of SK-OV-3 or MDA-MB-468 or combinations of these cells

**Software and algorithms**		

FlowJo Ver. 10	FlowJo LLC	https://www.flowjo.com/solutions/flowjo
ImageJ	U.S. National Institutes of Health	https://imagej.nih.gov/ij/download.html
Inveon Research Workplace software	Siemens	https://www.siemens-healthineers.com/br/molecular-imaging/preclinical-imaging/preclinicalglobal-support
Prism Ver. 9 for MacOS	GraphPad Software, Inc.	https://www.graphpad.com/

**Other**		

Positron Emission Tomography System	Siemens	MicroPET Focus 220
Computed Tomography System	GE Healthcare	eXplore Locus Ultra Preclinical CT
Microplate Reader	Biotek	Cytation Gen 5
Flow Cytometer	Beckman-Coulter	CytoFLEX S

### Resource availability

#### Lead contact

Further information and requests for resources and reagents should be directed to and will be fullfilled by the Lead Contact, Raymond M. Reilly at the University of Toronto; raymond.reilly@utoronto.ca.

#### Materials availability

This study did not generate new unique reagents. [^64^Cu]Cu-NOTA-trastuzumab Fab-PEG_24_-EGF bsRICs and monospecific [^64^Cu]Cu-NOTA-trastuzumab Fab or [^64^Cu]Cu-NOTA-EGF are not available but may be synthesized according to the published method.^17^ There are restrictions to the availability of these radiopharmaceutical agents due to Canadian Nuclear Safety Commission (CNSC) regulations on the transfer of radioactive materials. SK-OV-3 and MDA-MB-468 cells and NOD-SCID mice for tumor engraftment are available commercially. All other methods and data are described in the article and further information is available from the lead contact upon reasonable request.

#### Data and code availability

• All data reported in this paper will be shared by the lead contact upon request.
• This paper does not report original code.
• Any additional information required to reanalyze the data reported in this paper is available from the lead contact upon request.

### Experimental model and study participant details

#### Monospecific and bispecific radioimmunoconjugates

[^64^Cu]Cu-NOTA-trastuzumab Fab-PEG_24_-EGF bispecific radioimmunoconjugates (bsRICs) and monospecific [^64^Cu]Cu-NOTA-trastuzumab Fab or [^64^Cu]Cu-NOTA-EGF were synthesized and characterized as previously reported.^17^

#### Flow cytometry measurement of HER2-positive and EGFR-positive cells

SK-OV-3 human female ovarian carcinoma cells with high HER2 (9.6 × 10^5^ HER2/cell) but low EGFR (8.0 × 10^4^ EGFR/cell) expression^20^ and MDA-MB-468 human female breast cancer cells with high EGFR (1.3 × 10^6^ EGFR/cell)^21^ but negligible HER2 [American Type Culture Collection (ATCC)] were cultured in Dulbecco’s Modified Eagle medium (Gibco) supplemented with 10% FBS (5% CO_2_ at 37°C). Cells were tested annually for mycoplasma. The cell doubling times were determined by culturing 2.0 × 10^4^ cells in 24-well plates and measuring the number of viable cells every 24 h up to 96 h using an automated cell counter (Millipore). Mixtures of cells (total = 1.0 × 10^6^ cells) consisting of SK-OV-3 (100%, 75%, 50%, 25%, 0%) and MDA-MB-468 cells (0%, 25%, 50%, 75%, 100%) were cultured for 24 h, then characterized for HER2-positive and EGFR-positive cells. Cells were recovered into phosphate-buffered saline (PBS) and immunostained with anti-EGFR Alexa Fluor 647 conjugated panitumumab (1 μg per 5.0 ×10^5^ cells; ThermoFisher) or anti-HER2 Alexa Fluor 488 conjugated trastuzumab (1 μg per 5.0×10^5^ cells) for 1 h at 4°C. Cells were rinsed 4 times with PBS with 1% BSA and analyzed by flow cytometry (CytoFLEX S; Beckman-Coulter) using two channels: 660 nm emission peak/20 nm band filter and 525 nm emission peak/40 nm band filter. Results were analyzed with FlowJo Software Ver. 10 (FlowJo LLC).

#### Tumor xenograft mouse models

Tumor xenografts with homogeneous or heterogeneous expression of HER2 or EGFR were established in four weeks old female NOD-SCID mice by s.c. inoculation of 5 × 10^6^ human SK-OV-3 ovarian cancer cells (100%) or MDA-MB-468 cells (100%) into the left flank or a mixture of SK-OV-3 cells (30%) and MDA-MB-468 cells (70%) into the right flank. The proportion of SK-OV-3 and MDA-MB-468 cells selected to establish heterogeneous tumors was based on *in vitro* measurement of the cell doubling times, such that the tumor *in vivo* would express both HER2-positive and EGFR-positive cells at 6 weeks post-inoculation. At this time point, mice were sacrificed and the tumors excised and dissociated into a single cell suspension using FBS-free medium containing 0.1 mg/mL Collagenase Type 1 (Sigma-Aldrich). The cells were immunostained as before with Alexa Fluor 647 conjugated panitumumab or Alexa Fluor 488 conjugated trastuzumab and analyzed by flow cytometry. *Ex vivo* immunohistochemical (IHC) staining of tumors was performed to assess the spatial distribution of EGFR and HER2 expression. Tumors were embedded into formalin-fixed, paraffin blocks and 10 μm sections were immunostained at the Pathology Research Program Laboratory at the University Health Network for EGFR using rabbit anti-human EGFR monoclonal antibodies (Cell Signaling Technology, Product #4267) and HER2 using rabbit anti-human HER2 monoclonal antibodies (ThermoFisher/Invitrogen, Product #SP3). Digital brightfield images of each slide were acquired using a Cytation Gen5 microplate reader (BioTek). The percentage of HER2-positive or EGFR-positive stained cells was estimated by the ImmunoRatio plugin in ImageJ software (NIH). ImmunoRatio utilizes the color deconvolution algorithm which segments immunostained and brown diaminobenzidine (DAB)-stained cellular areas. All animal studies were conducted under an Animal Care Protocol (AUP282.16) approved by the Animal Care Committee at the University Health Network following Canadian Council on Animal Care (CCAC) guidelines.

### Method details

#### PET/CT imaging and biodistribution studies

[^64^Cu]Cu-NOTA-trastuzumab Fab-PEG_24_-EGF specifically binding to both HER2 and EGFR or monospecific [^64^Cu]Cu-NOTA-trastuzumab Fab or [^64^Cu]Cu-NOTA-EGF monospecific for HER2 or EGFR, respectively were synthesized and characterized as reported.^17^ Groups of NOD-SCID mice (*n* = 5) with s.c. tumors on the right flank homogeneous for HER2 or EGFR, or on the left flank heterogeneous for HER2 and EGFR were injected i.v. (tail vein) with 15–20 MBq (10 μg) of these bispecific or monospecific agents. Mice were sedated with isoflurane 2% in oxygen and imaged at 24 h and 48 h post-injection (p.i.) on a PET tomograph (Siemens MicroPET Focus 220). Images (supine position) were acquired for 20–60 min then were reconstructed by filtered back-projection and ordered subset expectation maximization methods, followed by a maximum *a posteriori* probability reconstruction algorithm with no correction for attenuation or partial-volume effects. The full-width-at-half-maximum resolution of the PET tomograph was 1.37 mm in the transaxial plane at the center of the field of view. Immediately after PET imaging, mice were transferred to an eXplore Locus Ultra Preclinical CT Scanner (GE Healthcare) for a whole-body CT scan using routine acquisition parameters (16 s anatomical scan at 80 kVp, 50 mA, and voxel size of 150 × 150 × 150 μm. MicroPET and CT images were co-registered using Inveon Research Workplace software (Siemens). Immediately after the 48 h imaging time point, mice were sacrificed and tumors and samples of blood and normal tissues were collected, weighed and ^64^Cu measured in a γ-counter. Tumor and normal tissue uptake were expressed as percentage injected dose per gram (%ID/g).

### Quantification and statistical analysis

Statistical comparisons were made by Student’s t test (*p* < 0.05) using Prism Ver. 9 for MacOS (GraphPad Software, San Diego, CA) and are shown in footnotes in Tables 1 and 2 and in Figure 4D. The number of replicate determinations is indicated by “n”.

### Additional resources

None.
